# Le profil lipidique et glucidique des accidents vasculaires cérébraux ischémiques à Dakar

**DOI:** 10.11604/pamj.2016.25.29.8906

**Published:** 2016-09-27

**Authors:** Cisse Ousmane, Dadah Samy Mohamed Lemine, Ba Fatoumata, Ba El Hadji Makhtar, Diop Marieme Soda, Diagne Ngor Side, Sow Adjaratou Dieynaba, Basse Anna Modji, Touré Kamadore, Ndiaye Moustapha, Diop Amadou Gallo, Ndiaye Mouhamadou Mansour

**Affiliations:** 1 Clinique Neurologique du Centre Hospitalier Universitaire de Fann-Dakar, Sénégal; 2 Centre Neuropsychiatrique de Nouakchott, Mauritanie; 3 Laboratoire de Physiologie UGB de Saint Louis, Sénégal; 4 Service de Médecine Préventive et de Santé Publique de la FMPO de Dakar, Sénégal

**Keywords:** Accident vasculaire cérébral ischémique, glycémie, bilan lipidique, Sénégal, Ischemic cerebrovascular accident, blood glucose, lipidic profile, Senegal

## Abstract

L’accident vasculaire cérébral (AVC) se définit comme le développement rapide de signes cliniques localisés ou globaux de dysfonction cérébrale sans autre cause apparente qu’une origine vasculaire. Différents facteurs de risque ont été identifiés et associés à la survenue des AVC ischémiques dont les perturbations du métabolisme glucidique et lipidique. Nous avons mené une étude rétrospective à la clinique neurologique de Fann. Notre étude a porté sur les dossiers des patients hospitalisés du 1er Janvier au 31 Décembre 2010 pour un AVCI confirmé par l’imagérie. Tous nos patients avaient beneficié d’un bilan lipidique complet (cholestérol total, triglycérides, HDL ; le taux de LDL ayant été calculé grâce à la formule de Friedwald, d’un bilan rénal et d’une glycémie à jeun prélevés dans les 48 heures suivant l’admission. Les données ont été analysées en mesure univariée, ensuite bivariée grace au logiciel SPSS 16.0. Nous avons colligé 235 dossiers. Les patients étaient âgés de 10 à 99 ans avec une moyenne à 67,06 ans. Le sexe masculin était à 42,55%, le sex-ratio était de 0,74 en faveur des femmes. Vingt Six pour cent avaient une glycémie à jeun anormale à la phase aiguë de l’AVCI. Le bilan lipidique montrait une augmentation du cholestérol total chez 52,34% des patients. Le HDL était bas chez 34,47% des patients. L’hypertriglycéridémie n’avait été observée que chez 3% des patients. Le LDL était élevé chez 12,76%. L’indice d’athérogénicité était élevé chez 25,53% des patients. Des perturbations de la glycémie et du bilan lipidique sont très souvent associées à l’AVCI et doivent être prises en compte pour assurer une meilleure prévention secondaire.

## Abstract (en)

Cerebrovascular accident (CVA) is defined as the rapid development of localized or global clinical signs of neurological dysfunction with no apparent cause other than that of vascular origin. A variety of risk factors have been identified and associated with the occurrence of Ischemic CVA, including glucose and lipid metabolism disturbances. We conducted a retrospective study at the Clinic of Neurology, Fann. Our study focused on medical records of patients with ICVA confirmed by imaging, hospitalized from January 1 to December 31 2010. All patients underwent complete lipid profile (total cholesterol, triglycerides, HDL; LDL level was calculated using Friedwald formula), kidney function tests and fasting blood sugar test were performed within 48 hours of admission. Data were analysed using univariate technique and then using bivariate technique tanks to SPSS 16.0 software. We collected 235 files. We here report a case series of patients between ages 10-99 years, with an average age of 67,06 years. Males were 42,55%, sex-ratio was 0,74 in favour of women. 26% of cases had impaired fasting glucose levels during the acute phase of ICVA. The lipid profile showed an increase in total cholesterol level in 52.34% of patients. Low levels of HDL cholesterol were found in 34.47% of patients. Hypertriglyceridemia was only observed in 3% of patients. LDL levels were high in 12,76% of patients. Atherogenicity index was high in 25,53% of patients. Disturbances of blood glucose and lipid profile are often associated with ICVA and should be taken into account to ensure better secondary prevention.

## Introduction

L’accident vasculaire cérébral (AVC) se définit comme le développement rapide de signes cliniques localisés ou globaux de dysfonction cérébrale sans autre cause apparente qu’une origine vasculaire [1]. C’est une affection touchant le plus souvent le sujet âgé et fortement invalidante dans les pays développés : 1ère cause d’handicap physique acquis de l’adulte. Différents facteurs de risque sous-tendus par des déterminants socio-économiques, culturels, politiques et environnementaux ont été identifiés et associés à la survenue des AVC ischémique dont les perturbations du métabolisme glucidique et lipidique. Plusieurs études sur la prévention primaire et secondaire de ces facteurs ont été menées et la qualité de la prise en charge a été améliorée ces dernières années. Notre étude a pour objectif spécifique la description du profil lipidique et glucidique observé chez des victimes d’accidents vasculaires cérébraux ischémiques de même que leur impact sur la survie et la récupération fonctionnelle des patients atteints.

## Méthodes

Il S’agit d’une étude retrosopective menée à la clinique neurologique du centre hospitalo-universitaire de Fann (Dakar-Sénégal) portant sur les dossiers de patients hospitalisés pour AVCI (confirné par l’imagerie). Elle s’étendait du 1er Janvier au 31 Décembre 2010. Tous nos patients avaient beneficié dans les 48H suivant l’admission, d’un bilan lipidique complet (cholestérol total, triglycérides, HDL ; le LDL ayant été calculé grâce à la formule de Friedwald), d’un bilan rénal et d’une glycémie à jeun. Les variables sociodémographiques, les données cliniques et biologiques ont été étudiés en mesure univarié. Ensuite une analyse bivarié correlant les aspects biologiques aux différentes données ont été réalisées. Les analyses étaient réalisées par le biais du logiciel de SPSS 16.0.

## Résultats

Nous avons collecté 235 dossiers de patients. L’age moyen de nos patients était de 67,06 ans avec un écart-type de 13,89 ans et des extrémes allant de 10 à 99 ans. Les tranches d’âge les plus représentatives étaient celles comprises entre 65 à 74 ans et entre 75 à 84 ans respectivement à 28,94% et 28,51%. Le sex- ratio était de 0,74 en faveur des femmes (57,45%). L’hypertension artérielle et le diabète étaient les antécédents médicaux les plus fréquents avec respectivement 88,94% et 15,74%? on note que 14,89% des patients étaient à la fois hypertendus et diabétiques.

La glycémie variait entre 0,39g/l et 2,42 g/l avec une moyenne totale de 1,02g/l et un écart-type de 0,32. 67,6 % des patients avaient une glycémie entre 0,7 et 1,1g/l, 8,5% avaient une glycémie entre 1,26-2g/l (Voir Tableau 1).

**Tableau 1:** Valeurs de la glycémie

	Fréquence	Pourcentage
<0,7 g/l	15	5,3
0,7 - 1,1 g/l	195	68,9
1,1- 1,25g/l	37	13,1
1,26- 2g/l	24	8,5
>2g/l	10	3,5
Total	281	99,3
System	2	,7
Total	283	100,0

Les formes de dyslipidémies observées par ordre décroissant de fréquence dans notre étude étaient l’hypercholestérolémie totale, l’hypocholestérolémie à HDL, l’hypercholestérolémie à LDL et l’hypertriglycéridémie. Le bilan lipidique montrait un taux de cholestérol total moyen de 1,78g a été observé avec des extrêmes de 0,75g à 3,54g /l. Il avait été observé une augmentation du cholestérol total chez 52,34% des patients. Chez les hommes, le taux moyen de cholestérol obtenu était de 1,65 g /l contre 1,88 g /l chez les femmes. Un taux bas de HDL était rencontré chez 34,47% des patients (Voir Tableau 2). La valeur du LDL variait avec des extrêmes de 0,058 g/l et 2,866 g/l avec une moyenne générale de 1,08 g/l avec un taux de1,06 g/l chez les hommes et de 1,10 g/l chez les femmes. 12,76% avaient un taux de LDL élevé. L’indice d’athérogénicité calculé avec la formule cholestérol total/HDL cholestérol était élevé c’est-à-dire supérieure à 4,85 chez (25,53%) des patients.

**Tableau 2:** Valeurs des paramètres lipidiques

	Valeurs minimales (g/l)	Valeurs maximales (g/l)	taux d’anormalité (%)	Moyenne générale (g/l)	Moyenne chez les hommes (g/l)	Moyenne chez les femmes (g/l)
Cholestérol Total	0,75	3,54	52,34	1,78	1,65	1,88
Triglycérides	0,12	3,59	2,98	0,71	0,77	0,63
Taux de HDL	0,13	1,59	34,47	0,55	0,46	0,62
Taux de LDL	0,058	2,866	12,76	1,08	1,06	1,10

Parmi les patients qui avaient une hyperglycémie anormale à la phase aigüe de l’AVCI, 27 décès(44,26%) avaient été observés contre 34,15% pour ceux ayant une hypercholestérolémie totale, 34,57% pour un taux de HDL bas.

## Discussion

Dans notre série, l’âge moyen était de 67,06 ans avec une prédominance féminine. Cette tendance a également été au Gabon où 56,2% des patients étaient des femmes [2]. Une étude menée par Hollander et al [3] a révélé également une nette prédominance féminine à 58.4%. L’HTA est le facteur de risque principal de l’AVCI, elle serait associée à 56% des AVCI chez l´homme et 66% chez la femme en Europe selon l’étude de L’ESI en 2001 [4]. Nos résultats sont comparables à ceux de Chan [5] qui avait trouvé 84% d’HTA, supérieur à ceux de Sweileh [6] à 69,9% et de Sène Diouf [7] à 63,53 %. En dehors de l’HTA, le diabète est également un facteur de risque fréquemment associé aux AVC et multiplie le risque relatif d´AVCI par 1,5 à 3. Ce risque relatif est plus élevé chez la femme à 2,2 contre 1,8 chez l´homme [8]. Notre taux est inferieur à celui de Chan [5] et de Sweileh [6] où les taux étaient respectivement de 21 % et 45,2%. Nos résultats sont similaires à ceux de Sène Diouf [7] et de Ducluzeau [9] avec respectivement 11,76 et 11%.

La prévalence de l’hyperglycémie dans notre étude est de 25,96%. Il est à noter que la répartition des patients présentant une hyperglycémie suivant la tranche d’âge montre une prédominance dans celles de 65 à 84 ans; où les AVCI sont plus fréquents. Ceci suggère le rôle prédominant de l’hyperglycémie dans la survenue des AVCI. Ces données sont identiques à celle de Apetse au Togo [10] et à Ashok en Lybie [11], avec une prédominance féminine de l’hyperglycémie. Plusieurs études ont montré que les taux de mortalité à court et à long terme et le risque de décès étaient plus élevés chez les patients victimes d´AVC avec une hyperglycémie à l’admission [11–17].

Dans une revue systématique de l´hyperglycémie de stress et le résultat de l’accident vasculaire cérébral, Capes et al [18] ont rapporté une mise en commun de 2 fois plus de risque de mortalité à court terme (moins de 1 mois) suivant 3 études ayant fourni des données pour les patients diabétiques et non diabétiques combinés. Williams et al [19] dans une étude de 656 patients hospitalisés pour un AVCI ont constaté que l´hyperglycémie à l’admission était présente dans 40% des cas et indépendamment augmentait le risque de mortalité à 30 jours, 1 an et 6 ans. Kiers et al [14] ont observé que la mortalité était significativement plus élevée chez les patients présentant une hyperglycémie de stress et qu’ils ont également tendance à présenter un tableau plus grave. Nos résultats sont semblables à cet égard.

Dans notre série, la dyslipidémie représente un facteur plus fréquent devant le diabète mais une étude cas-témoins est nécessaire pour apprécier son impact dans la survenue des AVCI. Certaines études ont rapporté les mêmes données [12, 20]. La prédominance féminine a été rapportée par Ashok et al [11]. Depuis 1992 grâce à une méta-analyse anglaise, il est établi que le risque relatif d´AVC en cas d´hypercholestérolémie est de 1,3 à 2,9 et que la suppression de l´hypercholestérolémie permettrait d´éviter 22 000 AVC par an parmi les sujets de plus de 55 ans en Angleterre [13, 18].

L’étude Multiple Risk Factor Intervention (MRFIT) [21] a démontré une relation significative entre le cholestérol sérique et le risque d’AVCI et une relation inverse avec les AVC hémorragiques. Les mêmes constatations ont été faites au cours de l’étude Copenhague City Heart [22]. Dans l´étude de la population à Hisayama [23], l´association globale entre la concentration LDL-cholestérol et l’AVC n´était pas significative. Cependant, l´incidence ajustée pour l´âge, le sexe, le type d’AVCI a montré de façon significative des niveaux croissants de LDL. En comparaison avec le premier quartile de la distribution de LDL, les individus dans le quatrième quartile étaient plus à risque d´infarctus cérébral athérothrombotique (HR) 2,84, IC 1,17 -6 95% • 93) [23].

Une revue systématique récente a constaté que les concentrations de HDL-cholestérol ont été inversement associées au risque d´AVCI et d’athérosclérose carotidienne [24]. Dans une revue systématique combinant neuf études prospectives, il a été constaté que la concentration de triglycérides était un prédicteur important de tous les AVC (RR 1,10, 1,07 -1 • 13, p <0,0001), sans hétérogénéité entre les études (p = 0,96) [25]. Les résultats de l’étude Copenhague Heart City Study [22] avaient indiqué une association convaincante entre les concentrations de triglycérides et le risque d’AVCI. La raison pour laquelle l´hétérogénéité dans la cause de l´AVC n´affecterait pas l´association entre le taux de cholestérol HDL ou des concentrations de triglycérides et le risque d´AVC, mais aurait une incidence sur l´association entre le taux de cholestérol LDL ou le cholestérol total et le risque d´AVC n´est pas claire. Les études récentes MIRACL, TNT, ASCOT ont également mis en évidence une diminution nette du risque d´AVC lorsque le traitement des dyslipidémies était efficace.

## Conclusion

Notre étude confirme l’importante prévalence des troubles du métabolisme glucidique et lipidique associés aux AVCI. Ces troubles du métabolisme doivent être recherchée systématiquement car leur présence aggrave l’atteinte cérébrale de ces patients et détériore leur pronostic fonctionnel et vital. Le traitement doit prendre en compte l’altération de ces paramètres biologiques pour assurer une meilleure prise en charge et prévention secondaire plus efficace.

### Etat des connaissances actuelles sur le sujet

- La dyslipidémie est un facteur de risque cardiovasculaire;
- L’hypoglycémie et l’hyperglycémie sont néfastes pour le cerveau;
- Le LDL cholestérol est plus délétère que le HDL cholestérol.

### Contribution de notre étude à la connaissance

- La prévalence des troubles du métabolisme glucidique et lipidique chez les patients victimes d’accident vasculaire cérébral ischémique;
- Ces troubles aggrave l’atteinte cérébrale de ces patients et détériore leur pronostic fonctionnel et vital.
